# Effects of a taped filter mask on peak power, perceived breathlessness, heart rate, blood lactate and oxygen saturation during a graded exercise test in young healthy adults: a randomized controlled trial

**DOI:** 10.1186/s13102-022-00410-8

**Published:** 2022-02-07

**Authors:** Hoi Lam Ng, Johannes Trefz, Martin Schönfelder, Henning Wackerhage

**Affiliations:** Exercise Biology Group, Department of Sport and Health Science, Technical University of Munich, Connollystraße 32, 80809 Munich, Germany

**Keywords:** SARS-CoV-2, COVID-19, Masks, Exercise test

## Abstract

**Background:**

Face masks are an effective, non-pharmacological strategy to reduce the transmission of Severe Acute Respiratory Syndrome Coronavirus-2 and other pathogens. However, it is a challenge to keep masks sealed during exercise, as ventilation can increase from 5 to 10 L/min at rest to up to 200 L/min so that masks may be blown away from the face. To reduce leakage e.g. during exercise, a face mask was developed that is taped onto the face. The aim of this study was to investigate during a graded exercise test the effect of a taped filter mask on the perception of breathlessness, heart rate, blood lactate concentration, and oxygen saturation when compared to a surgical mask and no mask.

**Methods:**

Eight healthy trained participants (4 females), aged 24.5 ± 3.3 years performed graded exercise test until volitional exhaustion under three conditions: (1) No mask/control, (2) surgical mask or (3) taped filter mask. During these tests, we measured perception of breathlessness, heart rate, blood lactate concentration and peripheral oxygen saturation and analysed the resultant data with one or two-way repeated measures ANOVAs. We also used a questionnaire to evaluate mask comfort and analysed the data with paired t-tests.

**Results:**

When compared to wearing no mask, maximal workload was significantly reduced with a taped filter face mask by 12 ± 6% (*p* < 0.001) and with a surgical mask by 3 ± 6% (*p* > 0.05). Moreover, subjects perceive the sensation of "severe breathlessness" at a 12 ± 9% lower workload (*p* = 0.012) with a taped face mask, and 7 ± 13% lower workload with a surgical mask (*p* > 0.05) when compared to wearing no mask. Oxygen saturation at 65% of the maximal workload is 1.5% lower (*p* = 0.018) with a taped mask than no mask. Heart rate and blood lactate concentration are not significantly different in-between no mask, surgical mask and taped mask at any workload. When compared to wearing a surgical mask, wearing a taped filter face mask has a significantly better wearing comfort (*p* = 0.038), feels better on the skin (*p* = 0.004), there is a lower sensation of moisture (*p* = 0.026) and wearers perceive that less heat is generated (*p* = 0.021). We found no sex/gender differences for any of the measured parameters.

**Conclusions:**

A taped mask is well tolerated during light and moderate exercise intensity but reduces maximal exercise capacity.

**Supplementary Information:**

The online version contains supplementary material available at 10.1186/s13102-022-00410-8.

## Background

Since December 2019, the global coronavirus disease 2019 (COVID-19) pandemic has fundamentally changed the way we live, travel and exercise [1–3]. By the end of January 2022, there were 373 million confirmed cases of COVID-19 globally, and over 5.6 million deaths have been recorded [4]. COVID-19 is caused by the Severe Acute Respiratory Syndrome Coronavirus-2 (SARS-CoV-2) which is a respiratory virus that can be transmitted through droplets, aerosol and SARS-CoV-2-contaminated surfaces [5, 6]. Breathing, speaking, coughing and sneezing can produce droplets and aerosols [7–9] and SARS-CoV-2 can remain active for hours in aerosol [10].

The COVID-19 pandemic and measures to contain its spread have also affected exercise, sport and sporting events [2]. In relation to this, the arguably most consequential decision was to postpone the 2020 Tokyo Olympic Games to 2021. This was the first time in history that Olympic Games were cancelled due to a health issue [11]. In addition, governments banned competitive sports and closed sporting facilities such as gyms and swimming pools. Not only did this negatively affect the sports economy but it also altered physical activity levels [2].

Face masks are an effective, non-pharmacological strategy to reduce SARS-CoV-2 transmissibility and infection [12–19]. Face masks can also prevent infections during sport, as Watson et al. [20] reported that the use of face masks was associated with decreased COVID-19 incidence during sports. The question arises, however, whether different types of face masks have negative effects on ventilation, cardiovascular function and metabolism. This issue has been addressed in publications that have measured the effects of different types of face masks on hemodynamic, cardiopulmonary, and metabolic parameters during exercise [18, 21–30]. Some studies found that wearing a face mask during exercise reduced cardiopulmonary function or exercise performance [9, 24, 27, 28]. However, other studies showed that wearing a face mask does not hinder performance or cardiorespiratory function [21, 22, 29, 30]. These diverse results may result from different study designs and methods, such as constant or progressive load protocols.

A limitation of conventional face masks is that air may increasingly escape unfiltered when ventilation increases from 5 to 10 L per minute (L/min) at rest to over 100 L/min in untrained subjects [31] and up to 200 L/min e.g. in highly trained rowers [32–34]. Air leakage from the sides of face masks can decrease mask efficacy and is likely to increase the risk of droplet transmission [35]. To address the issue of leakage of unfiltered air at high levels of ventilation, a taped filter mask was developed, where a “filtering facepiece 2” (FFP2) filter is taped onto the face with a liminar KinesioTape.

Here, we investigate during graded exercise tests the effect of this taped filter mask on the perception of breathlessness, oxygen saturation, heart rate, blood lactate concentration and exercise performance during a graded exercise test when compared to a surgical mask and no mask. Specifically, we aimed to answer three research questions [36]:

1. Up to what intensity can subjects exercise with a taped filter mask without the perception of major breathlessness and/or a stable drop of the peripheral capillary oxygen saturation (SpO_2_) to below 80%?
2. How does a taped filter mask affect heart rate and the concentration of blood lactate during a graded exercise test when compared to a surgical mask and no mask?
3. How comfortable is a taped filter mask during a graded exercise test when compared to a surgical mask?

## Methods

This study was approved by the ethical committee of the Technical University of Munich (Approval No. 584/20 S). Eight trained and healthy subjects were recruited and completed the study (Table 1). All participants were informed about the experimental procedures, requirements, and risks prior to the start of the study. All participants signed an informed consent form voluntarily and were informed that they can withdraw from the study at any time without consequence. A total of nine participants were invited, eight participants agreed to take part in the study and all of them fit our study inclusion criteria. Participants recruited were university students or from nearby sports clubs. Subject recruitment was done via flyers, notices, and online media such as websites and the “System for recruiting test subjects” (“System zur Probandenrekrutierung”) of the Faculty of Sport and Health Sciences (TUM). Inclusion criteria of participants included age between 18 and 50 years old, normal weight with body mass index (BMI) between 19 and 25 kg/m^2^, regular endurance training of at least 3 h per week. Exclusion criteria were cardiovascular, pulmonary, neurological, metabolic, chronic disease or other conditions that prevents one to perform an endurance test such as injury. Pregnant females were excluded. Individuals with history of suspected or confirmed doping test were not included. Also, individuals who smoked in the last 12 months were excluded.

**Table 1:** Physical characteristics of participants

	All (N = 8)		Male (N = 4)		Female (N = 4)	
	Mean ± SD	Range	Mean ± SD	Range	Mean ± SD	Range
Age (years)	24.5 ± 3.3	19–30	25.3 ± 3.6	22–30	23.8 ± 3.2	19–26
Height (cm)	178 ± 13	152–192	187 ± 3.4	184–192	169 ± 12.5	152–182
Weight (kg)	74.9 ± 9.9	62–88	82 ± 7.5	71–88	67.8 ± 6.2	62–76
BMI (kg/m^2^)	23.6 ± 2.4	20.5–27.3	23.4 ± 2.3	20.5–26	23.9 ± 2.8	20.8–27.3

*N* number, *SD* standard deviation, *cm* centimeter, *kg* kilogram, *BMI* body mass index, *m*^*2*^ square metre

**Fig. 1:**
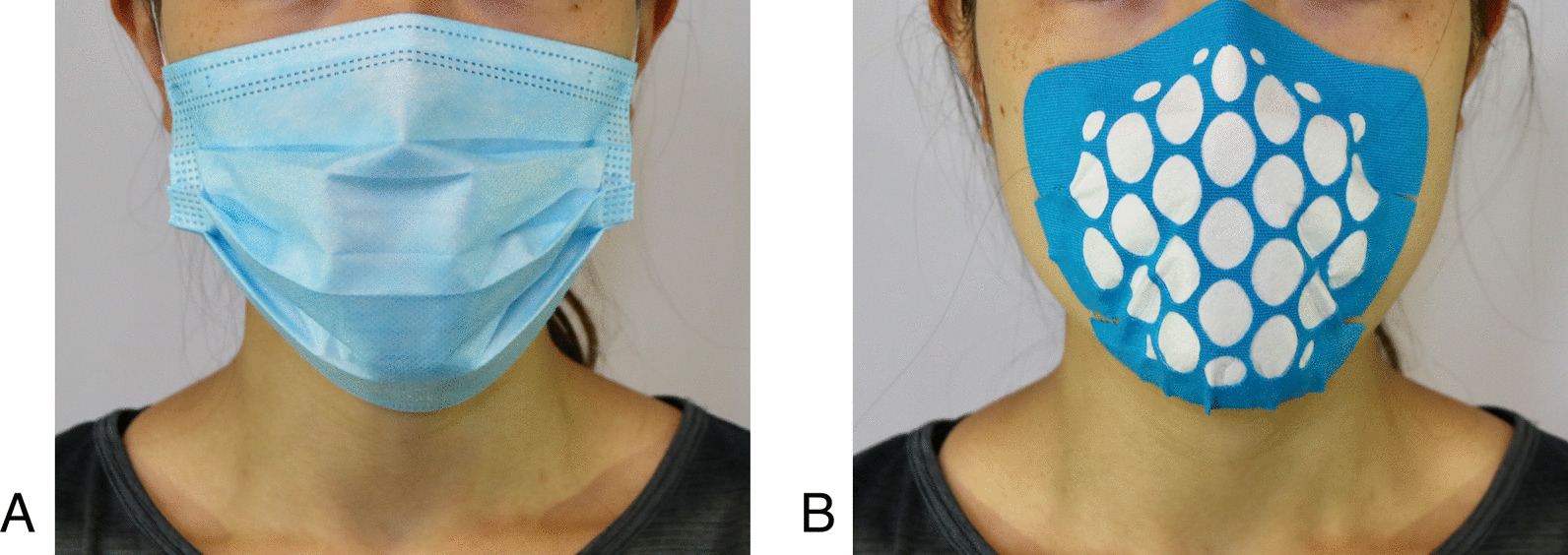
Photos of participant wearing **A** a surgical mask and **B** a taped filter mask

The study had three study conditions:

1. No mask control,
2. Commercially available surgical face mask (Fig. 1A) and a
3. Taped filter mask (Fig. 1B).

The characteristics of the two face masks are described in Table 2. For a good fit of taped filter masks, male participants in our study used the larger size, whereas female participants used the smaller size. The taped masks were provided by TRC The Rendering Company GmbH (TRC).

**Table 2:** Physical characteristics of the masks

Mask type	Size (cm)	Product name Materials	Thickness (mm)
Taped mask	15 × 12 16 × 12.8	The proper mask Adhesive carrier layer is comparable to KinesioTape (Suzhou MedSport Products Co., LTD.) and is combined with a two-layer polypropylene filter material complying with FFP2 (EN149) norm (Textilmacher GmbH; Munich, GER)	0.46
Surgical mask	17.5 × 9.5	Moon-Valley face mask Non-woven fabric (70%), Melt-blown fabric (30%)	0.4

*cm* centimetre, *mm* millimetre

### Outcome variables

**Fig. 2:**
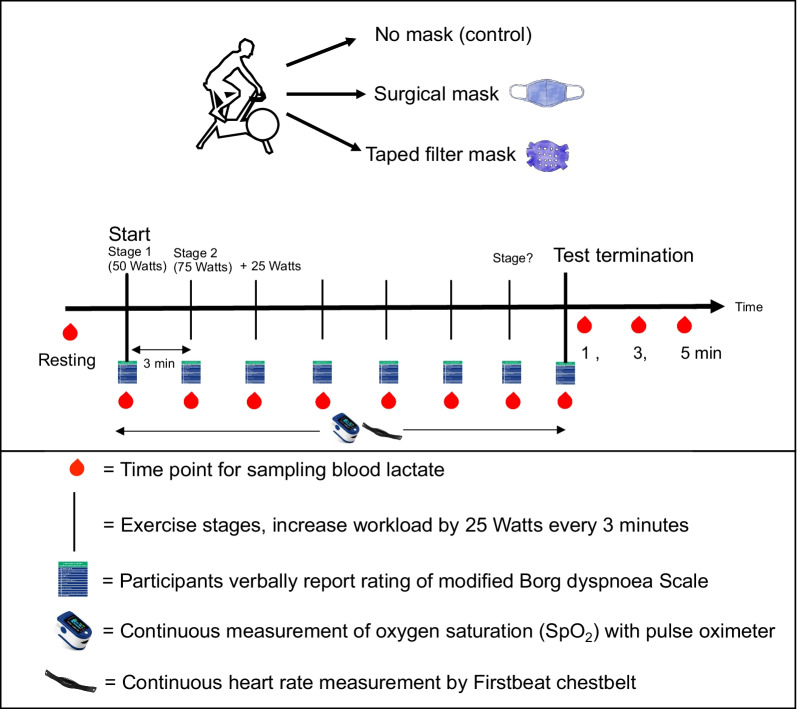
Illustration of the experiment conditions and measuring time points for the outcome variables

The measured parameters included perception of breathlessness (dyspnoea scale), heart rate, SpO_2_ and blood lactate concentration which we will explain in the following sections (Fig. 2).

### Dyspnoea (shortness of breath) rating

Subjects were asked to verbally rate the dyspnoea that they perceived at the end of each stage from a rating of 0–10, using the modified Borg dyspnoea scale [37] (Table 3). A copy of the scale including the ratings and their corresponding breathlessness intensities were presented at all times for the participant during the graded exercise test so that the participants could take reference from it. We arbitrarily defined dyspnoea ratings equal or above 7 (very severe) in the modified Borg dyspnoea scale as major breathlessness.

**Table 3:** Modified Borg dyspnoea scale [37]

Rating	Corresponding breathlessness intensity
0	No shortness of breath at all
0.5	Very, very light (barely perceptible)
1	Very light
2	Mild
3	Moderate
4	Rather severe
5	Severe
6	
7	Very severe
8	
9	Very, very severe
10	Maximal respiratory distress

### Heart rate

Study participants wore a heart rate chest belt (Firstbeat Technologies Oy; FI) which continuously detects heart rate with a sampling frequency of 1 Hz throughout the graded exercise test. The use of Firstbeat device to monitor heart rate has been validated [38]. We used the Firstbeat SPORTS software tool (Version 4.7.3.1) to record the heart rate measurement. For the evaluation we determined the mean value of the detected signals of the last 30 s of each stage.

### Oxygen saturation (SpO_2_)

Oxygen saturation (SpO_2_) was measured continuously by a pulse oximeter on a fingertip (Nonin 8000A, USA) with a sampling frequency of 5 Hz. Pulse oximetry is a non-invasive method to detect the differential absorption of light from oxygenated and deoxygenated haemoglobin, and thus estimates the percentage of haemoglobin-bound oxygen saturation in the arterial blood [39, 40]. We used the Lode Ergometry Manager software (version 10.6.0) to record oximeter data.

### Blood lactate concentration

To measure the metabolic response to exercise, 20 µl of capillary blood was taken from the earlobe of the subjects to determine the blood lactate concentration. Blood was collected once at rest, at the end of each exercise stage, upon termination of the test, and at 1, 3, and 5 min after the end of the test. Subsequently, we determined the lactate values amperometrically using Biosen S Line device (EKF; GER), which has been used by other studies [41, 42]. The lower detection limit of the instrument was a blood lactate concentration of 0.5 mmol/L.

### Mask questionnaire

After completing the graded exercise test wearing a surgical mask or a tape filter mask, study participants provided feedback on their subjective well-being and evaluation of the masks during the test by answering a questionnaire. The content of the questionnaire was modified from previous studies [24, 43] (Table 4). The questionnaire recorded subjective ratings and qualitative information about the comfort and wearability of the taped filter mask and surgical mask during the exercise test. Using this questionnaire, the subjects evaluated 10 sentences on a visual analogue scale ranging from 0 (totally disagree) to 10 (totally agree) and provided comments in an additional field.

**Table 4:** Content of the mask questionnaire

Question	Statement
1	Your overall condition is very good today
2	The facemask prevents you from your maximal performance
3	The facemask fits very well
4	The wearing comfort of the facemask is very good
5	The facemask material feels very good on the skin
6	The sensation of moisture with the facemask is very low
7	The sense of smell with the facemask is very good
8	Breathing is very difficult with the facemask
9	The heat generated with the facemask is very low
10	Suitable for intensive sports activities

Modified after [24, 43]

### Study protocol

All participants underwent a medical fitness examination at the start of the study to ensure they had no contraindication to exercise testing and to check for inclusion criteria. After passing medical screening, subjects participated in three graded exercise tests on three different days. All three tests were performed at the same time of day, within a range of two hours. Minimum time of recovery between the single test was 24 h. No specific diet or fluid intake instruction was given to the participants prior to the testing. No specific instruction was given to avoid exercise before a certain timeframe of the test and participants were asked to maintain their normal weekly routine. For each testing session, participants performed a graded cycling test on a Lode Excalibur bicycle ergometer (Lode, B.V.; NL) until voluntary exhaustion. During these three tests, subjects either wore no mask (control), a surgical mask or a taped filter mask. To avoid potential serial effects, the order of three mask conditions were randomized. The exercise protocol began at a workload intensity of 50 watts (W) and each 3 min, the workload increased by 25 W until exhaustion [44]. We used the Lode Ergometry Manager software (version 10.6.0) to set the protocol and control for the increasing load intensities during the graded exercise test. The test was terminated either during volitional exhaustion, or until subjects reached level 10 on the dyspnoea scale or a stable SpO_2_ < of 80% that has been used in other studies as evidence for critical hypoxemia [45, 46].

### Statistics

A sample size estimation was done before the commencement of the study and this study was powered to detect a 5% difference in maximum workload, assuming the peak power output would be 250 W during the exercise test. For statistical evaluation, we used JASP software tool (version 0.12.0) to conduct the statistical analysis. As the duration of three exercise tests varied across conditions, for appropriate analysis of our exercise variables (perception of breathlessness, heart rate, blood lactate concentration and oxygen saturation) with a comparable exercise intensity, data was normalized, interpolated, and expressed in relative to individual peak power achieved (percentage of peak power) in the control condition. The exercise variables were assessed initially by one-way ANOVA for repeated measures to compare between the three conditions. For SpO_2_, heart rate and blood lactate concentration, one-way repeated measures ANOVAs were also done at each normalised workload in a 5%-interval (e.g. at 0% peak power, 5% peak power etc.) to identify any statistical significance between groups at various workloads. If there was a significant main effect, we used Bonferroni post-hoc tests for multiple comparisons.

To examine if there were differences between the ratings for surgical mask and taped filter mask from the mask questionnaire, paired t-tests were done for each domain of the questionnaire. For secondary analysis, we added gender as a between-group factor and conducted two-way ANOVA for repeated measures to evaluate if there was a gender difference in all analyses. Significance level was set as *p* < 0.05 for all statistical tests.

## Results

Eight participants completed three graded exercise tests each with no adverse effects that required ending an experiment prematurely. Our participants reached 95 ± 3% of their maximal predicted heart rate and a rating of 9.7 ± 0.5 out of 10 of the modified Borg dyspnoea scale when they finished the exercise test. Table 5 shows the main results of the graded exercise test. We now state and answer the three research questions of this study.

**Table 5:** Results of the graded exercise test

Parameters	NM	SM	TFM	ANOVA	NM versus SM	NM versus TFM	SM versus TFM
Maximal workload (Watt)	278 ± 56	269 ± 56	247 ± 56	** < 0.001**	0.560	** < 0.001**	**0.018**
Time to exhaustion (minutes)	29.2 ± 6.6	27.5 ± 6.3	25.6 ± 6.2	** < 0.001**	**0.046**	** < 0.001**	**0.046**
Maximal dyspnoea rating	9.8 ± 0.5	9.7 ± 0.7	9.8 ± 0.5	0.935	1.000	1.000	1.000
Minimal SpO_2_%	92 ± 3	90 ± 6	89 ± 4	0.211	1.000	0.249	1.000
Maximal heart rate (beat per minute)	189 ± 5	185 ± 3	182 ± 7	0.066	0.330	0.069	0.330
Maximal Lactate concentration (mmol/L)	9.0 ± 2.0	7.8 ± 1.4	6.6 ± 2.0	**0.011**	0.164	**0.009**	0.164

Results of the parameters are reported as mean ± standard deviation

*NM* no mask, *SM* surgical mask, *TFM* taped filter mask, *ANOVA* analysis of variance, *SpO*_*2*_ oxygen saturation, *La* blood lactate concentration

*p* Values are shown for statistical analysis

Significant results are indicated in bold

### Up to what intensity can healthy, trained subjects exercise with a taped filter mask without the perception of major breathlessness and/or a drop of the oxygen saturation (SpO_2_) to below 80%?

**Fig. 3:**
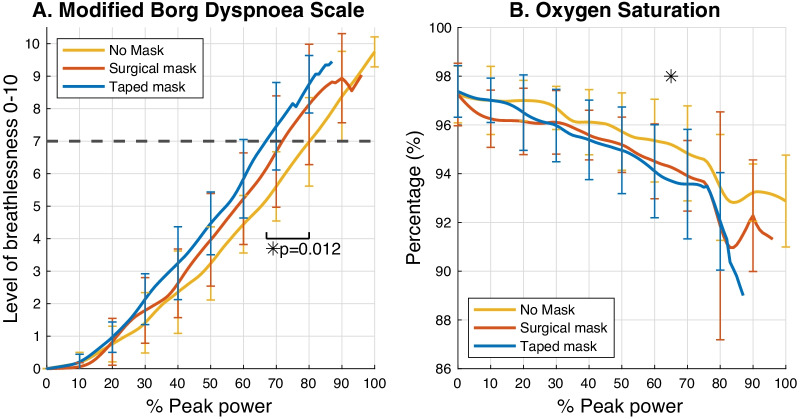
Ratings of the modified Borg dyspnoea scale (**A**) and oxygen saturation (**B**) during graded exercise tests. Data are expressed relative to the maximal workload achieved in the control (no mask) condition. Black horizontal dotted line in **A** indicates the breathlessness threshold of 7 (very severe). One-way repeated measures ANOVAs were done for each parameter at each normalised workload in a 5%-interval. We performed statistical tests until 90% of peak power because in some trials the subjects did not reach this percentage of their maximal workload. Therefore there was not enough data for statistical analysis. Significant findings are marked with a "*"

Our results shows that participants reached a rating of major breathlessness (i.e. a rating of “7”) at 68 ± 7% of peak power with the taped filter mask, 73 ± 11% with a surgical mask and 80 ± 10% in the control trial with no mask (Fig. 3A). Overall, when comparing with wearing no mask, participants reached major breathlessness at 12 ± 9% lower workload (*p* = 0.012) with a taped filter mask and 7 ± 13% lower workload (*p* > 0.05) with a surgical mask. Participants reached major breathlessness earlier with a taped filter mask than a surgical mask at 5 ± 7% lower workload but this was not statistically significant (*p* > 0.05). When gender is added for a between-group factor for secondary analysis with two-way repeated measures ANOVA, no significant differences were found in-between gender (F(1,6) = 1.362, *p* = 0.288) or for a mask × gender interaction (F(2,12) = 0.163, *p* = 0.852). The only significant effect was the effect of the mask where subjects with a taped filter mask reached major breathlessness earlier than when wearing no mask (*p* = 0.021). Specifically, males rated dyspnoea as 7 (very severe) at 65 ± 7% of peak power with the taped filter mask, at 69 ± 10% with a surgical mask and 78 ± 12% in the control trial with no mask, respectively. Females reached a rating of 7 (very severe) at 71 ± 7% with the taped filter mask, 78 ± 12% with the surgical mask and 82 ± 7% of peak performance with no mask, respectively.

Overall, participants’ SpO_2_ values at 65% of the maximal workload were significantly lower by 1.5% when wearing the taped filter mask than when wearing no mask (*p* = 0.018) (Fig. 3B). In general, SpO_2_ responses to exercise varied greatly in-between subjects, and they dropped to below 85% in two subjects. Only in one participant did the saturation drop below 80% but recovered within seconds and reached 92% at the end of the test. For these reasons, the test was not aborted. There were no statistically significant differences for minimal SpO_2_ values between the three mask conditions (F(2,14) = 1.742, *p* = 0.211), gender (F(1,6) = 0.898, *p* = 0.380) or mask × gender interaction (F(2,12) = 0.367, *p* = 0.700). The mean minimal values were 92 ± 3% without wearing a mask, 90 ± 6% when wearing the surgical mask and 89 ± 4% when wearing the taped filter mask.

### How does a taped filter mask affect heart rate and the concentration of blood lactate during a graded exercise test when compared to a surgical mask and no mask?

**Fig. 4:**
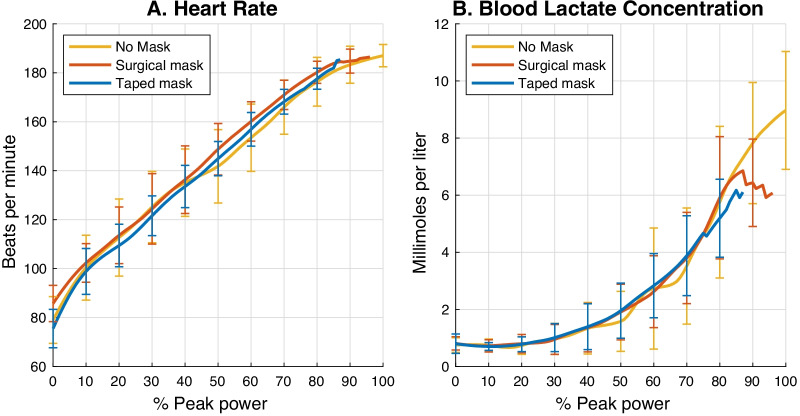
Heart rate (**A**) and blood lactate concentration (**B**) during graded exercise tests. Heart rate data from all three tests of two participants were excluded due to technical error. The peak blood lactate concentrations were 9 ± 2 mmol/L with no mask, 7.8 ± 1.4 mmol/L with surgical mask and 6.6 ± 2.0 mmol/L when wearing a taped filter mask (Main effect F(2,14) = 6.356, *p* = 0.011), respectively. Post-hoc analyses reveal that the maximal blood lactate concentration reached with a taped filter mask is significantly lower than that reached with no mask (*p* = 0.009)

Figure 4 shows the average heart rate and blood lactate concentration during the three exercise conditions, expressed relative to the maximal workload achieved in the no-mask test. There were no significant differences in the heart rate and blood lactate concentration at any workload (*p* > 0.05), but subjects achieved the highest blood lactate concentrations in the test where they did not wear a face mask. Table 6 shows the blood lactate concentration after exercise.

**Table 6:** Post-exercise blood lactate concentrations

	No mask (mmol/L)	Surgical mask (mmol/L)	Taped filter mask (mmol/L)	ANOVA	No mask versus surgical mask	No mask versus taped filter mask	Surgical mask versus taped filter mask
Post 1 min	9.0 ± 2.0	7.5 ± 1.7	6.4 ± 1.9	**0.012**	0.111	**0.011**	0.188
Post 3 min	8.0 ± 2.2	7.2 ± 1.1	6.0 ± 2.4	**0.040**	0.256	**0.039**	0.239
Post 5 min	7.6 ± 2.2	6.6 ± 0.9	5.3 ± 2.8	**0.019**	0.202	**0.017**	0.202
Δ Post1–End	1.0 ± 1.0	0.6 ± 1.4	0.9 ± 1.2	0.797	1.000	1.000	1.000
Δ Post3–Post1	− 0.9 ± 0.5	− 0.2 ± 1.0	− 0.5 ± 1.3	0.448	0.667	0.803	0.803
Δ Post5–Post3	− 1.5 ± 1.9	− 0.7 ± 0.9	− 0.7 ± 0.6	0.377	0.670	0.670	0.965

Results of the blood lactate concentration are reported in mean ± standard deviation. mmol/L = millimoles per liter. ANOVA = Analysis of variance. Δ Post1-End = Change between post 1 min measurement and measurement at test termination. Δ Post3-Post1 = Change between post 3 min measurement and post 1 min measurement. Δ Post5-Post3 = Change between post 5 min measurement and post 3 min measurement

*p* Values are shown for statistical analysis

Significant results are indicated in bold

### How comfortable is a taped filter mask during a cycling ergometer test when compared to a surgical mask?

**Fig. 5:**
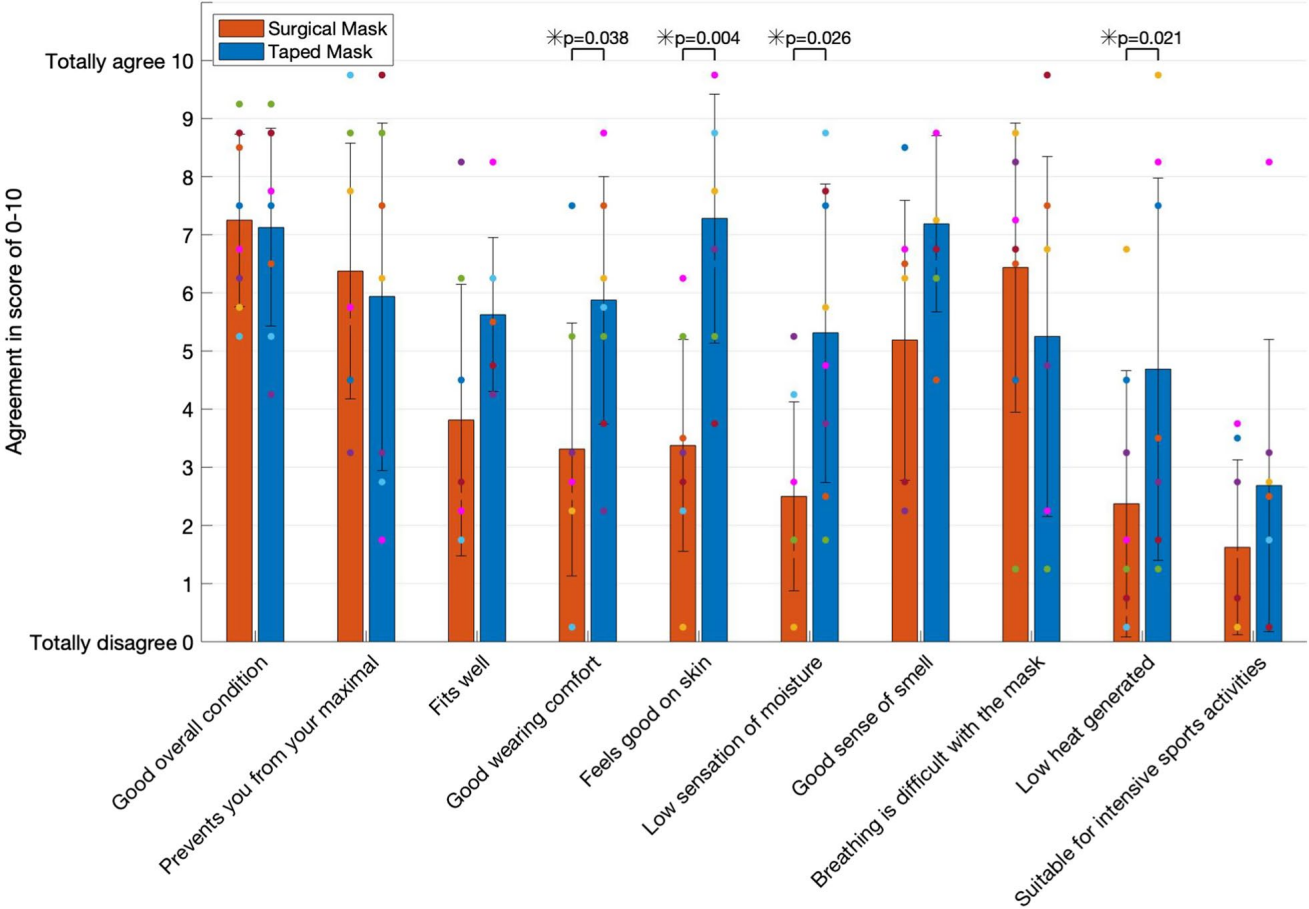
Results of the mask questionnaire. Data presented with means (bars), standard deviations (error bars) and individual ratings (colour dots) of the 10 statements

When compared to wearing a surgical mask, subjects felt that the taped filter masks were more comfortable (*p* = 0.038), felt better on the skin (*p* = 0.004), reduced the sensation of moisture (*p* = 0.026) and subjects had a reduced perception of heat (*p* = 0.021) during the graded exercise test (Fig. 5). For all the other variables, no significant differences were found in-between conditions (*p* > 0.05).

In five out of eight participants the taped filter mask partially detached during vigorous exercise [47] when the corresponding heart rate was 89 ± 4% of the maximal predicted heart rate. Typically, these were small gaps between the adhesive tape and skin after 20.4 ± 3.3 min at 210 ± 22 W. Qualitative feedback provided by the participants are listed in Additional file 1.

## Discussion

The main finding of this study is that a taped filter mask significantly reduces the maximal workload in a graded exercise test by 12 ± 6% when compared to no mask. This is no surprise as a well-sealed, taped filter mask will increase breathing resistance. However, a taped filter mask is tolerated well during mild to moderate exercise [47] and was more comfortable to wear than a surgical mask.

### Perceived breathlessness and drop in oxygen saturation

Participants wearing a taped filter mask perceived major breathlessness (subjectively defined as a dyspnoea scale rating of 7 or above) earlier, at a 12 ± 9% lower exercise intensity when compared to no mask. Our participates reached major breathlessness at 68 ± 7% of peak power with the taped filter mask, which corresponded to reaching 84 ± 5% of the maximal predicted heart rate during vigorous intensity [47]. This might result from the potentially higher breathing resistance (not measured) because of the better sealing of the taped mask. Our results are comparable to those of Wong et al. [9] who reported an increase of rating of perceived exertion (RPE) when they walked for 6 min at 4 km per hour (km/h) when wearing a surgical mask compared to no mask. However, Epstein et al. [22] found no difference in the RPE between no mask, surgical mask and N95 respiratory during graded exercise test on cycle ergometer. This may be explained by the different study protocols [22] as their tests started at 25 W and used a ramp protocol by increasing 25 W every 3 min. They also used a non-invasive nasal prong throughout the tests which may have added breathing resistance to the control/no mask condition.

We found, however, no statistical difference in minimal SpO_2_ during the exercise tests. One male subject transiently reached a SpO_2_ of 75% at 83% of the maximal workload while wearing a surgical mask. However, the SpO_2_ recovered quickly and rose to 92% at the end of the test. We continued the test as we interpreted this brief decline to below 80% as a technical error. Precision and accuracy of SpO_2_ readings can be affected by technical problems, poor perfusion (e.g. a cold finger) or environmental factors such as movement artefacts [39, 48]. Whilst other studies found no differences between no mask and masks in terms of oxygen saturation [22, 29, 30] or partial pressure of oxygen [24] during exercise, we observed a significantly lower SpO_2_ at 65% of the maximal workload when compared to no mask. However, the SpO_2_ drop was only 1.5% which should be only a minor difference physiologically (SpO_2_ at 65% was no mask: 95.2 ± 1.8%, surgical mask 94.3 ± 1.4%, taped mask 93.7 ± 1.9%).

### Heart rate and blood lactate concentration

We found no significant difference in the heart rate or blood lactate concentration at any exercise intensity from rest to 90% of the maximal workload achieved in the no mask test. This is consistent with other studies that compared wearing face masks to no mask during graded exercise tests [22, 29, 30]. Two studies [9, 27], however, reported significantly higher heart rates when exercisers wore a surgical mask than when not wearing a mask. Wong et al. [9] required healthy adults with a sport background to perform 6-min walking on treadmill (4 km/h) and Lässing et al. [27] conducted 30-min constant-load aerobic exercise on a cycle ergometer at individual’s maximal lactate steady state in healthy young men. Also, Li et al. [43] measured a higher heart rate in healthy adults when wearing N95 face mask than surgical mask during a total of 50-min walking in various intensities, ranging from 3.2 to 6.4 km/h. Different subjects, exercise modalities, face masks and intensities may explain these differences.

Subjects reached a higher peak blood lactate concentration when wearing no mask. This is primarily explained by the higher workload that the subjects achieved when wearing no mask which is consistent with another study [24]. For the post-exercise blood lactate concentration, the differences between test termination, Post 1, 3 and 5 min did not differ between groups. The result is consistent with the findings of Lässing et al. [27] in which they measured the recovery phase after a constant load exercise with or without surgical mask.

### Mask comfort

Subjects that wore a taped filter mask felt more comfortable, the taped filter mask felt better on the skin and had a reduced sense of moisture or heat from the mask during exercise when compared to wearing a surgical mask. In another study, Li et al. [43] compared the subjective sensations of N95 and surgical face masks during intermittent exercise on a treadmill. They found that participants felt that the surgical mask was drier, cooler, easier to breathe and more comfortable when compared to a N95 mask. Our study is the first to test the comfort of a taped filter mask.

In some of the experiments, the taped mask loosened partially and there were gaps between the tape and the skin. This could be a result of the movement of the mouth and nose because of deep breathing in combination with sweating. To ensure good tape adhesion, male users should therefore shave their face before exercise, and participants should avoid makeup or skin creams. We observed a poor mask adhesion in two male participants at their chin. It is unclear whether this was due to beard growth or because of the shape of the face. Some participants commented that the taped face mask is suitable for light and moderate exercise but not during maximal exercise.

### Limitations

We did not measure ventilation as a spirometry mask would have affected moisture build-up and thereby the property of face masks worn below a spirometry mask. Testing with a spirometry mask would also reduce external validity of our experiments. Also, we had a small sample size since we only investigated 4 males and 4 females which performed three tests each in randomised order. Furthermore, we arbitrarily defined the criterion for major breathlessness and the questionnaire that we used to assess subjective mask comfort is not validated. Lastly, we did not control the room temperature and relative humidity.

## Conclusions

In summary, taped filter masks are tolerated well during mild and moderate exercise but they reduce maximal exercise capacity when compared to no mask. Subjects reported that they are more comfortable to wear during exercise on a cycling ergometer than a surgical mask, and they are a potential new tool to avoid becoming infected by SARS-CoV-2 or other infectious agents at rest and during exercise. Future studies may investigate the use of such taped filter masks on other activities such as fitness training in a gym, during sports such as martial arts and in occupations that require well sealing, comfortable face masks. Future variants of taped face masks could have a stronger adhesive e.g. for martial arts or long-duration sports and may include technical solutions to allow drinking during sport.

## Supplementary Information

**Additional file 1**: Qualitative feedback from mask questionnaire. In this table it listed participants’ feedback on the surgical and taped mask during exercise.

## Data Availability

The datasets used and/or analysed during the current study are available from the corresponding author on reasonable request.

## Abbreviations

COVID-19: Coronavirus disease 2019
SARS-CoV-2: Severe acute respiratory syndrome coronavirus-2
L/min: Liter per minute
FFP2: Filtering facepiece 2
SpO_2_: Peripheral capillary oxygen saturation
SD: Standard deviation
Cm: Centimetre
kg: Kilogram
m^2^: Square metre
Mm: Millimetre
W: Watts
NM: No mask
SM: Surgical mask
TFM: Taped filter mask
La: Blood lactate concentration
ANOVA: Analysis of variance
RPE: Rate of perceived exertion
Km/h: Kilometre per hour
mmol/L: Millimoles per liter

## References

[1] Abdullah M, Dias C, Muley D, Shahin M (2020). Exploring the impacts of COVID-19 on travel behavior and mode preferences. Transp Res Interdiscip Perspect..

[2] Wackerhage H, Everett R, Krüger K, Murgia M, Simon P, Gehlert S (2020). Sport, exercise and COVID-19, the disease caused by the SARS-CoV-2 coronavirus. Dtsch Z Sportmed.

[3] Huang C, Wang Y, Li X, Ren L, Zhao J, Hu Y (2020). Clinical features of patients infected with 2019 novel coronavirus in Wuhan, China. The Lancet.

[4] WHO. WHO Coronavirus (COVID-19) Dashboard 2021 [updated 1 June 2021; cited 31 January 2022].

[5] CDC. Ways COVID-19 Spreads 2020 [updated 28 October 2020; cited 25 March 2021].

[6] WHO. Coronavirus disease (COVID-19). How is it transmitted? 2020 [updated 20 October 2020; cited 25 March 2021].

[7] Bahl P, Doolan C, De Silva C, Chughtai AA, Bourouiba L, MacIntyre CR. Airborne or droplet precautions for health workers treating COVID-19? J. Infect. Dis.. 2020. 10.1093/infdis/jiaa189 PMC7184471 32301491

[8] Kähler CJ, Hain R (2020). Flow analyses to validate SARS-CoV-2 protective masks.

[9] Wong AYY, Ling SKK, Louie LHT, Law GYK, So RCH, Lee DCW (2020). Impact of the COVID-19 pandemic on sports and exercise. Asia-Pac J Sports Med Arthrosc Rehabil Technol.

[10] van Doremalen N, Bushmaker T, Morris DH, Holbrook MG, Gamble A, Williamson BN (2020). Aerosol and surface stability of SARS-CoV-2 as compared with SARS-CoV-1. N Engl J Med.

[11] Dhillon M (2020). Olympics in the time of a pandemic. Indian J Orthop.

[12] Chu DK, Akl EA, Duda S, Solo K, Yaacoub S, Schünemann HJ (2020). Physical distancing, face masks, and eye protection to prevent person-to-person transmission of SARS-CoV-2 and COVID-19: a systematic review and meta-analysis. The Lancet.

[13] Howard J, Huang A, Li Z, Tufekci Z, Zdimal V, van der Westhuizen H-M (2021). An evidence review of face masks against COVID-19. Proc Natl Acad Sci..

[14] Leung NH, Chu DK, Shiu EY, Chan K-H, McDevitt JJ, Hau BJ (2020). Respiratory virus shedding in exhaled breath and efficacy of face masks. Nat Med.

[15] Santos-Silva PR, Greve JMDA, Pedrinelli A (2020). During the coronavirus (covid-19) pandemic, does wearing a mask improve or worsen physical performance?. Rev Bras Med Esporte.

[16] Wang Y, Tian H, Zhang L, Zhang M, Guo D, Wu W (2020). Reduction of secondary transmission of SARS-CoV-2 in households by face mask use, disinfection and social distancing: a cohort study in Beijing, China. BMJ Global Health.

[17] Doung-Ngern P, Suphanchaimat R, Panjangampatthana A, Janekrongtham C, Ruampoom D, Daochaeng N (2020). Case-control study of use of personal protective measures and risk for SARS-CoV 2 infection, Thailand. Emerg Infect Dis.

[18] Scheid JL, Lupien SP, Ford GS, West SL (2020). Commentary: physiological and psychological impact of face mask usage during the COVID-19 pandemic. Int J Environ Res Public Health.

[19] Abbott BW, Greenhalgh M, Clair SIS, Bush J. Making sense of the research on COVID-19 and masks. Abbott Lab Of Ecosystem Ecology. 2020.

[20] Watson A, Haraldsdottir K, Biese K, Goodavish L, Stevens B, McGuine T. The association of COVID-19 incidence with sport and face mask use in United States high school athletes. medRxiv. 2021. 10.4085/1062-6050-281-21 PMC9913054 34793596

[21] Chan NC, Li K, Hirsh J (2020). Peripheral oxygen saturation in older persons wearing nonmedical face masks in community settings. JAMA.

[22] Epstein D, Korytny A, Isenberg Y, Marcusohn E, Zukermann R, Bishop B (2021). Return to training in the COVID-19 era: The physiological effects of face masks during exercise. Scand J Med Sci Sports.

[23] Eroglu H, Okyaz B, Türkçapar Ü (2018). The effect of acute aerobic exercise on arterial blood oxygen saturation of athletes. J Educ Train Stud.

[24] Fikenzer S, Uhe T, Lavall D, Rudolph U, Falz R, Busse M (2020). Effects of surgical and FFP2/N95 face masks on cardiopulmonary exercise capacity. Clin Res Cardiol.

[25] Haraf RH, Faghy MA, Carlin B, Josephson RA (2021). The physiological impact of masking Is insignificant and should not preclude routine use during daily activities, exercise, and rehabilitation. J Cardiopulm Rehabil Prev.

[26] Hopkins SR, Dominelli PB, Davis CK, Guenette JA, Luks AM, Molgat-Seon Y (2021). Face masks and the cardiorespiratory response to physical activity in health and disease. Ann Am Thorac Soc.

[27] Lässing J, Falz R, Pökel C, Fikenzer S, Laufs U, Schulze A (2020). Effects of surgical face masks on cardiopulmonary parameters during steady state exercise. Sci Rep.

[28] Pifarré F, Zabala DD, Grazioli G, i Maura IdY (2020). COVID-19 and mask in sports. Apunts Sports Med.

[29] Shaw K, Butcher S, Ko J, Zello GA, Chilibeck PD (2020). Wearing of cloth or disposable surgical face masks has no effect on vigorous exercise performance in healthy individuals. Int J Environ Res Public Health.

[30] Ahmadian M, Ghasemi M, Nasrollahi Borujeni N, Afshan S, Fallah M, Ayaseh H, et al. Does wearing a mask while exercising amid COVID-19 pandemic affect hemodynamic and hematologic function among healthy individuals? Implications of mask modality, sex, and exercise intensity. Phys Sportsmed. 2021;1–12. 10.1080/00913847.2021.1922947 33902400

[31] Loe H, Steinshamn S, Wisløff U (2014). Cardio-respiratory reference data in 4631 healthy men and women 20–90 years: the HUNT 3 fitness study. PLoS ONE.

[32] Clark JM, Hagerman FC, Gelfand R (1983). Breathing patterns during submaximal and maximal exercise in elite oarsmen. J Appl Physiol.

[33] Di Paco A, Catapano GA, Vagheggini G, Mazzoleni S, Micheli ML, Ambrosino N (2014). Ventilatory response to exercise of elite soccer players. Multidiscipl Respir Med.

[34] Mazic S, Lazovic B, Djelic M, Suzic-Lazic J, Djordjevic-Saranovic S, Durmic T (2015). Respiratory parameters in elite athletes–does sport have an influence?. Rev Port Pneumol (Engl Edit).

[35] Dbouk T, Drikakis D (2020). On respiratory droplets and face masks. Phys Fluids.

[36] Glass DJ (2010). A critique of the hypothesis, and a defense of the question, as a framework for experimentation. Clin Chem.

[37] Büsching G (2009). Wenn die Luft wegbleibt. Physiopraxis.

[38] Bogdány T, Boros S, Szemerszky R, Köteles F (2016). Validation of the Firstbeat TeamBelt and BodyGuard2 systems. Magy Sporttudományi Szle.

[39] Ascha M, Bhattacharyya A, Ramos JA, Tonelli AR (2018). Pulse oximetry and arterial oxygen saturation during cardiopulmonary exercise testing. Med Sci Sports Exerc.

[40] Nitzan M, Romem A, Koppel R (2014). Pulse oximetry: fundamentals and technology update. Med Dev (Auckl, NZ).

[41] Ahrend M, Schneeweiss P, Theobald U, Niess AM, Krauss I (2016). Comparison of laboratory parameters of a mountain bike specific performance test and a simulated race performance in the field. J Sci Cycl.

[42] Martínez-Lagunas V, Hartmann U (2014). Validity of the Yo-Yo intermittent recovery test level 1 for direct measurement or indirect estimation of maximal oxygen uptake in female soccer players. Int J Sports Physiol Perform.

[43] Li Y, Tokura H, Guo Y, Wong A, Wong T, Chung J (2005). Effects of wearing N95 and surgical facemasks on heart rate, thermal stress and subjective sensations. Int Arch Occup Environ Health.

[44] Hollmann W. Höchst-und Dauerleistungsfähigkeit des Sportlers: spiroergometrische Beurteilung und Untersuchungsergebnisse von männlichen und weiblichen Personen des 1. bis 8. Lebensjahrzehnts: Barth; 1963.

[45] Slepushkin V, Musaeva M, Bestaev G, Musaeva P (2020). The frequency of critical incidents at perioperative period in smoking patients. Eurasian Union Sci.

[46] Jenkins S, Čečins N (2011). Six-minute walk test: observed adverse events and oxygen desaturation in a large cohort of patients with chronic lung disease. Intern Med J.

[47] ACSM (2018). American College of Sports Medicine's guidelines for exercise testing and prescription.

[48] Kacmarek R, Stoller J, Heuer A. Analysis and monitoring of gas exchange. Egan’s Fundamentals of Respiratory Care. 10th ed. Mosby. 2012. p. 398.

